# Temporal Modulation of Corticospinal Excitability by Repetitive Peripheral Magnetic Stimulation in Healthy Young Adults

**DOI:** 10.3390/brainsci16010105

**Published:** 2026-01-19

**Authors:** Rehab Aljuhni, Srinivas Kumar, Christina Sawa, Sangeetha Madhavan

**Affiliations:** 1 Department of Physical Therapy and Health Rehabilitation, College of Applied Medical Sciences, Majmaah University, Al Majma’ah, Riyadh 15341, Saudi Arabia; rehabaljuhani@mu.edu.sa; 2 Brain Plasticity Lab, Department of Physical Therapy, College of Applied Health Sciences, University of Illinois at Chicago, Chicago, IL 60612, USA; srinikumar62@gmail.com (S.K.); csawa99@gmail.com (C.S.)

**Keywords:** neuroplasticity, priming, rPMS, CSE, TMS, neurophysiology

## Abstract

**Background**: Repetitive peripheral magnetic stimulation (rPMS) delivers magnetic pulses to peripheral nerves and muscles, producing afferent input that can modulate corticospinal excitability (CSE). While the effects of rPMS on upper-limb muscles have been explored, its short-term effects on lower-limb CSE remain less understood. This study aimed to investigate the short-term effects of rPMS on CSE in the tibialis anterior (TA) muscle among healthy individuals. **Methods**: Twenty participants completed a repeated- measure, pre-post study. rPMS was applied to the non-dominant TA muscle at 10% above motor threshold for 15 min. CSE was assessed using transcranial magnetic stimulation (TMS), with measurements of motor evoked potential (MEP) amplitude, latency, and duration recorded at baseline, immediately after, 30 min, and 60 min post-stimulation. All analyses were conducted on clean datasets following removal of artifact-related outliers. **Results**: MEP amplitude showed a significant main effect of Side (*p* = 0.005), with greater values on the stimulated compared to the non-stimulated side. No significant main effects were found for Time (*p* = 0.351) or for the Side × Time interaction (*p* = 0.900). Descriptively, the largest increase in amplitude on the stimulated side was observed at 30 min post-stimulation (12% above baseline). MEP latency and duration showed no significant main or interaction effects. **Conclusions**: In conclusion, a single rPMS session applied to the TA produced a modest, side-specific increase in CSE lasting up to 60 min, as reflected in MEP amplitude. However, the absence of a significant time effect and perhaps non-optimized stimulation parameters limit the interpretation of sustained neuromodulatory effects. Future studies should examine optimal stimulation parameters and explore underlying mechanisms using measures such as the cortical silent period and interhemispheric inhibition.

## 1. Introduction

Repetitive peripheral magnetic stimulation (rPMS) is a non-invasive neuromodulation technique that involves the application of magnetic stimulation at the periphery to induce localized changes in neuromuscular function, with downstream effects on the motor cortex [1,2]. rPMS is typically administered using the repetitive transcranial magnetic stimulation (rTMS) stimulator. The mechanism involves generating a magnetic field that induces a directed electrical current strong enough to depolarize peripheral neuronal membranes, thereby initiating a muscle action potential [3]. This stimulation produces rhythmic muscle contraction and relaxation, activating proprioceptive afferents that can lead to changes in corticospinal excitability (CSE), by influencing excitatory interneuron mediated by glutamate circuits and synaptic plasticity, resembling long-term potentiation-like mechanisms [2,3,4].

Compared with other peripheral stimulation techniques (e.g., electrical stimulation), rPMS offers several practical advantages, including painless stimulation, with minimal skin impedance issues, which allows deeper peripheral structure stimulation. Reported adverse effects are generally mild and transient, most commonly including muscle fatigue or mild local discomfort during stimulation, which typically resolve once the stimulation is discontinued [4].

Recognizing the impact of rPMS on CSE is significant for neurorehabilitation. It not only deepens our understanding of brain function but also provides a valuable tool to harness neuroplasticity to facilitate motor learning and skill acquisition. Several studies have investigated the effect of rPMS on upper limb CSE in healthy adults [5,6,7]. One study found that a single rPMS session applied to the median nerve significantly increased both the peak amplitude of motor evoked potential (MEP) for the abductor pollicis brevis (APB) muscle and the steepness of the recruitment curve immediately after stimulation [6]. Two other studies documented increases in MEP recruitment curve, normalized MEPs, and intracortical facilitation of the flexor carpi radialis (FCR) and extensor carpi radialis (ECR) muscles after rPMS. Importantly, these changes were maintained for up to 2 h post-stimulation [5,7].

While our current understanding of the impact of rPMS on CSE is foundational, several gaps remain. First, most studies have focused on upper limb muscles, with relatively little investigation into how rPMS influences CSE in lower-limb muscles such as the tibialis anterior. Second, although some studies have tracked changes in CSE over time following rPMS, the time course of these effects in lower-limb muscles remains underexplored. Third, existing research has largely emphasized MEP amplitude as the primary outcome, with limited attention to additional parameters such as latency or duration, which may offer further insight into the neurophysiological effects of rPMS.

The present study addresses these gaps by examining rPMS-induced changes in CSE of the tibialis anterior (TA) muscle across multiple time points and by incorporating multiple MEP outcome measures. Characterizing the magnitude and temporal profile of CSE changes in healthy individuals will lay the groundwork for translating these findings to neurologically impaired populations, helping to optimize the use of rPMS as a neuromodulation tool.

Accordingly, this study aimed to explore short-term changes in CSE following rPMS applied to the TA muscle in healthy individuals. We used single-pulse transcranial magnetic stimulation (TMS) to measure CSE at baseline, immediately after rPMS, and at 30 and 60 min post-stimulation. We hypothesized that rPMS would facilitate CSE in the TA, primarily reflected by an increase in MEP amplitude and MEP duration, and reduced MEP latency, and that these effects would be presented immediately after the stimulation, persist for 30 and 60 min post-stimulation.

## 2. Materials and Methods

### 2.1. Subjects

This study employed a within-subject, repeated-measures pre–post design. Twenty healthy young adults were recruited to examine the immediate neurophysiological effects of stimulation under controlled conditions while minimizing confounding factors related to aging and pathology. Inclusion criteria were age 18 years or older, absence of neurological or cognitive deficits, and no current orthopedic or musculoskeletal injury affecting the lower limbs. Exclusion criteria were based on standard contraindications for TMS, including: a history of epilepsy or seizures, use of medications known to influence CSE, concussion within the past 6 months, skull abnormalities or fractures, unexplained recurrent headaches, implanted regulatory devices, and current pregnancy [8]. The study procedures were approved by the Institutional Review Board (protocol #2022-0600) at the University of Illinois at Chicago and conformed to the Declaration of Helsinki (2013 revision) during the study period of 2022 to 2023. Participants’ characteristics are presented in Table 1.

#### Study Design and Procedures

**Figure 1:**
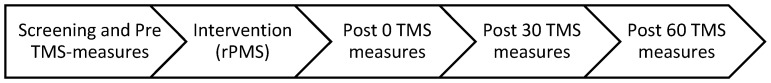
Flowchart of the study phases. Schematic diagram of the experimental timeline, showing baseline measurements, rPMS delivery, and subsequent post-stimulation assessments at each time point.

Participants attended the laboratory for a single visit. After providing written informed consent, they completed a screening questionnaire to determine eligibility. All procedures were conducted in a standardized, quiet laboratory environment under stable, controlled temperature and fixed lighting conditions for all participants to minimize external factors that could influence CSE. Eligible participants underwent baseline CSE measurements from both the dominant and non-dominant TA muscles. The TA muscle was selected because it is the primary ankle dorsiflexor and is located superficially, allowing for accurate surface electromyography (EMG) recordings and reliable assessment of CSE using TMS [9]. Leg dominance was determined based on self-report, defined as the leg preferred for kicking a ball [10]. rPMS was then applied to the non-dominant TA, and CSE was re-measured immediately after, 30 min after, and 60 min after stimulation on both sides. A schematic of the experiment timeline diagram is presented in Figure 1.

### 2.2. TMS Application

Participants were seated comfortably with knees bent at 90°, and the non-dominant foot was secured to a wooden board to resist dorsiflexion. EMG surface electrodes (Delsys, EMGworks, Natick, MA, USA) were placed over the midpoint of the TA muscle belly, with the reference electrode placed over the seventh cervical vertebra. Participants performed three maximum-effort ankle dorsiflexion trials (5 s each); the trial with the highest output was used to determine maximal voluntary contraction (MVC). The vertex was located as the intersection of the nasion–inion and preauricular lines. Single-pulse TMS was delivered using a 110 mm double-cone coil in a posterior–anterior orientation, connected to a Magstim 200 stimulator (Magstim Ltd., Wales, UK).

The TA “hotspot”—the scalp location that produced the largest and most consistent MEP at minimal intensity—was identified at baseline and marked on a fitted cap, and the same marked location and coil angle were used across all post-stimulation assessments [11]. Participants maintained a tonic contraction of the TA at 10% MVC during TMS. The active motor threshold (AMT) was defined as the lowest stimulator intensity that evoked MEPs greater than 50 µV in at least 4 out of 8 trials. Single pulse TMS was delivered at 120% of the individual AMT, consistent with prior research using suprathreshold stimulation to reliably elicit MEPs in healthy individuals [12].

### 2.3. rPMS Application

**Figure 2:**
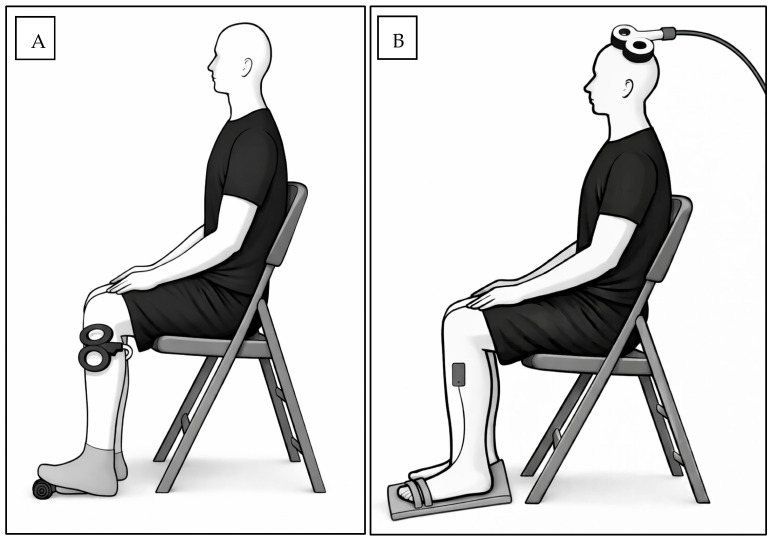
Schematic illustration of the experimental setups. (**A**) rPMS setup: Repetitive magnetic stimulation was delivered to the TA muscle, with a figure-of-eight coil positioned parallel to the muscle fibers. The TA muscle was maintained in a passively activated state using a small foot support. (**B**) TMS setup: A single-pulse magnetic stimulation delivered through a figure-of-eight coil positioned over the M1 area corresponding to the TA muscle. EMG electrodes were placed over the TA muscle, and the foot was stabilized on a wooden platform to maintain ankle position during recording.

rPMS was delivered to the non-dominant TA using an air-film-cooled figure-of-eight coil connected to a Magstim Rapid2 stimulator (Magstim, Wales, UK). The figure-of-eight coil is designed with double 70 mm coil windings to produce a biphasic focal magnetic field, and the integrated air-cooled system maintains performance during repetitive stimulation (Magstim Ltd., Wales, UK). A small support was placed under the non-dominant foot to maintain a slight isometric contraction of the TA muscle during stimulation. The TA muscle belly (located below the knee and lateral to the tibia) was identified and marked. The stimulation coil was positioned parallel to the muscle fibers over the marked site. AMT was determined via single-pulse trials and defined as the lowest stimulation intensity that elicited a visible or palpable contraction of the TA [5]. rPMS was administered at approximately 10% above AMT, using 2400 pulses delivered at 20 Hz over ~15 min. Stimulation was applied in 40 trains, each lasting 3 s with a 19 s inter-train interval [13]. The experimental setup for rPMS and TMS, including coil placement and EMG electrode positioning, is illustrated schematically in Figure 2.

### 2.4. Outcome Measure

#### Corticospinal Excitability (CSE)

CSE was assessed bilaterally at four time points: baseline, immediately post-rPMS, 30 min post-rPMS, and 60 min post-rPMS. At each timepoint, 20 single-pulse TMS trials were administered to record 20 MEPs from each TA, consistent with prior evidence demonstrating that 20–30 trials provide a reliable estimate of mean MEP amplitude and variability [14,15].

### 2.5. Data Analyses

**Figure 3:**
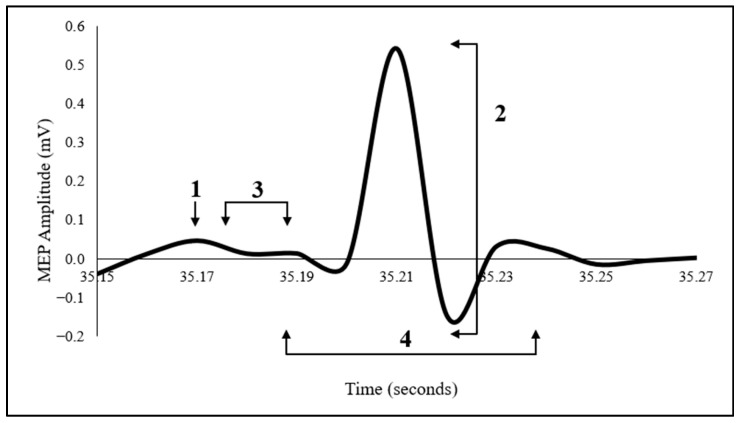
Representative motor evoked potential (MEP) waveform from the tibialis anterior muscle. Representative motor evoked potential (MEP) waveform recorded from the tibialis anterior muscle following single-pulse transcranial magnetic stimulation. The trace illustrates: (1) TMS trigger, (2) peak-to-peak MEP amplitude, (3) MEP latency, and (4) MEP duration.

EMG signals were bandpass filtered (10–500 Hz), sampled (2000 Hz), and amplified (1000×) using the default filtering settings provided by the EMG acquisition system (Delsys, Emgworks, MA, USA). Signals were stored for offline analysis in Spike2 (Cambridge Electronic Design, Cambridge, UK). To minimize electrode-skin impedance, the skin was shaved, and conductive gel was applied prior to surface EMG electrode placement. MEP parameters were defined as follows:

MEP amplitude: peak-to-peak was calculated as the voltage difference between the maximum positive and negative deflections within the post-stimulus window (in mV). MEP latency: defined as the time point at which the EMG signal visibly exceeded baseline activity following the TMS trigger (in ms). MEP duration: time between MEP onset and offset of the signal to baseline (in ms). A schematic figure of the MEP and its measurement is presented in Figure 3.

Data were analyzed using a custom automated script, followed by manual review for accuracy, with no additional baseline correction or noise-rejection. For each time point, average and standard deviation were computed across ~20 trials. Normalized MEP values were calculated as the percentage change from baseline and used in the final analysis [7]. Due to a hardware artifact affecting the TMS trigger signal, MEP latency data could not be reliably analyzed for two participants; therefore, latency analysis was conducted on 18 participants. As for outlier handling, it was performed in two steps: step 1—trial level, the 20 MEP recordings at each time point were screened and values exceeding ± 2 SD from the participant’s mean at each time point were removed; step 2—participant level, where mean values were then pooled, and any mean ± 2 SD from the group mean was excluded from the final analysis. Trials affected by EMG noise, stimulation artifacts, or signal loss were excluded prior to analysis. All remaining trials were used to compute outcome measures.

### 2.6. Statistical Analyses

A priori power analysis was conducted using G*Power software (version 3.1), based on effect sizes reported in a previous study [6]. Change in peak-to-peak MEP amplitude following rPMS was selected as the primary outcome measure. Assuming a 50% change in MEP amplitude, a sample size of 20 participants was estimated to provide 80% statistical power at an alpha level of 0.05. Accordingly, 20 healthy individuals were recruited. Data normality and homogeneity were assessed using the Shapiro–Wilk and Levene’s tests, respectively. Two-way repeated-measures ANOVAs were conducted for MEP amplitude, latency, and duration, with time (Post0, Post30, Post60) and side (stimulated, non-stimulated) as within-subject factors. When significant main effects or interactions were observed, post hoc pairwise comparisons were conducted using paired *t*-tests. For each dependent variable, percentage change from baseline was used for statistical analyses, while absolute values are reported descriptively. Mauchly’s test was used to assess sphericity, with Greenhouse–Geisser correction applied when violated.

Significance was set at *p* < 0.05. Effect sizes were reported as partial eta squared (ηp^2^), with benchmarks of 0.01 (small), 0.06 (medium), and 0.14 (large) [16]. Where applicable, 95% confidence intervals (CI) for effect sizes were also reported. All statistical analyses were performed using SPSS Statistics 31 (IBM, Armonk, NY, USA).

## 3. Results

Twenty participants completed the study (mean age = 25.5 years, SD = 5.40; 11 female). TMS was administered at baseline and at 0, 30, and 60 min after rPMS. After outlier handling, and due to outcome-specific artifact rejection and completeness. At the trial level, approximately 10% of trials (out of 20 per time point) were excluded following artifact inspection. At the participant level, four participants were excluded for amplitude, one for duration, and two for latency due to hardware artifact, yielding final samples of n = 16, 19, and 18, respectively. All inferential analyses were conducted on percentage change from baseline values, while absolute values are reported descriptively. Descriptive statistics for MEP amplitude, latency, and duration at each time point are presented in Table 2.

### 3.1. MEP Amplitude

Analyses were conducted on 16 participants. There was a significant main effect of side (F(1,15) = 10.908, *p* = 0.005, ηp^2^ = 0.421). Consistent with this finding, post hoc analysis using paired-samples *t*-tests comparing side-averaged values showed significantly higher scores for the stimulated side than the no-stimulated side (mean difference = 18.16, 95% CI [6.44, 29.87], t(15) = 3.30, *p* = 0.005) with a large effect size (Cohen’s d = 0.83, 95% CI [0.25, 1.39]).

**Figure 4:**
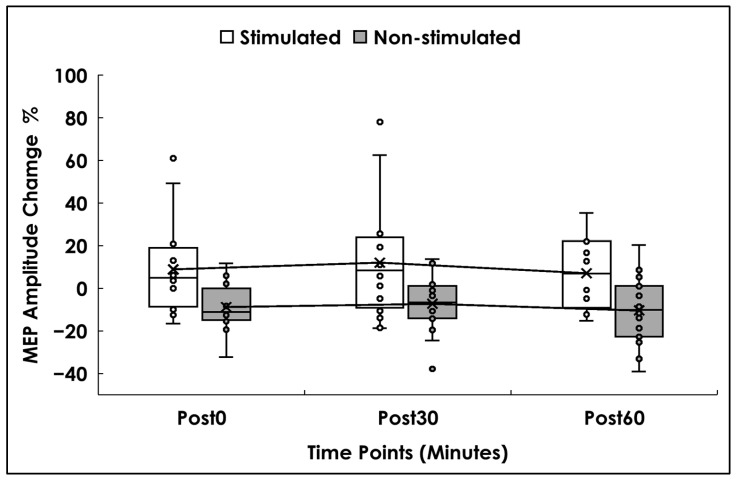
MEP Amplitude. Box and whisker plots represent the percentage change in MEP amplitude values (%), for the stimulated (white) and non-stimulated sides (gray) at 0 min (Post0), 30 min (Post30), and 60 min (Post60) after the stimulation. Small dots represent individual data, while the lower and upper horizontal lines represent minimum and maximum values. Boxes range from the 1st to the 3rd quartile, the X marks the mean values, and the middle horizontal lines represent median values. A mean line illustrates the trajectory of MEP amplitude across Post0, Post30, and Post60.

The main effect of time (F(2,30) = 1.085, *p* = 0.351, ηp^2^ = 0.067), and the time × side interaction (F(1.414, 21.212) = 0.050, *p* = 0.900, ηp^2^ = 0.003) were not significant. Greenhouse–Geisser correction was applied due to violation of Mauchly’s test (W = 0.586, *p* = 0.024). Descriptively, MEP amplitude on the stimulated side peaked at Post30 (12% above baseline), with a smaller increase at Post0 (9%), and Post60 (7%). In contrast, the non-stimulated side showed a decrease of (9%) at Post0, (7%) at Post30, and (10%) at Post60 (see Figure 4).

### 3.2. MEP Latency

**Figure 5:**
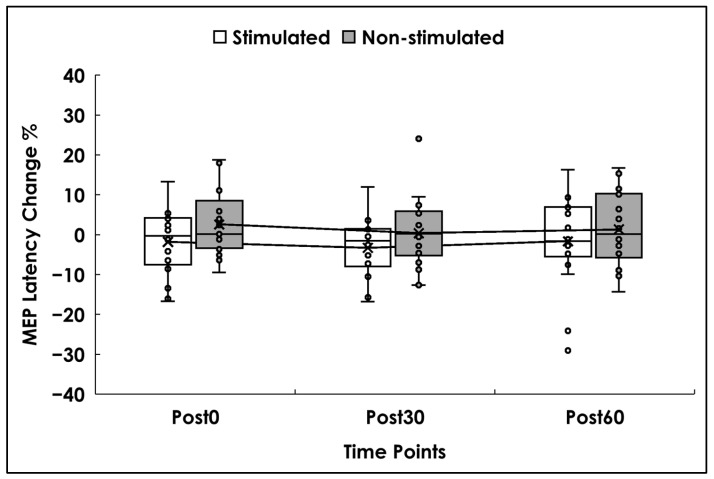
MEP Latency. Box and whisker plots represent the percentage change in MEP latency values (%), for the stimulated (white) and non-stimulated sides (gray) at 0 min (Post0), 30 min (Post30), and 60 min (Post60) after the stimulation. Small dots represent individual data, while the lower and upper horizontal lines represent minimum and maximum values. Boxes range from the 1st to the 3rd quartile, the X marks the mean values, and the middle horizontal lines represent median values. A mean line illustrates the trajectory of MEP latency across Post0, Post30, and Post60.

Analyses were conducted on 18 participants. One had missing latency data at Post0 for both sides, but their remaining data were included. There was no significant main effect of side (F(1,17) = 1.90, *p* = 0.188, ηp^2^ = 0.106), time (F(2,32) = 0.55, *p* = 0.582, ηp^2^ = 0.033), or the time × side interaction (F(2,32) = 0.15, *p* = 0.864, ηp^2^ = 0.009). Descriptively, latency on the stimulated side decreased slightly across time points (2% to 3%), whereas the non-stimulated side showed minimal increases (0.4–2%) (see Figure 5).

### 3.3. MEP Duration

**Figure 6:**
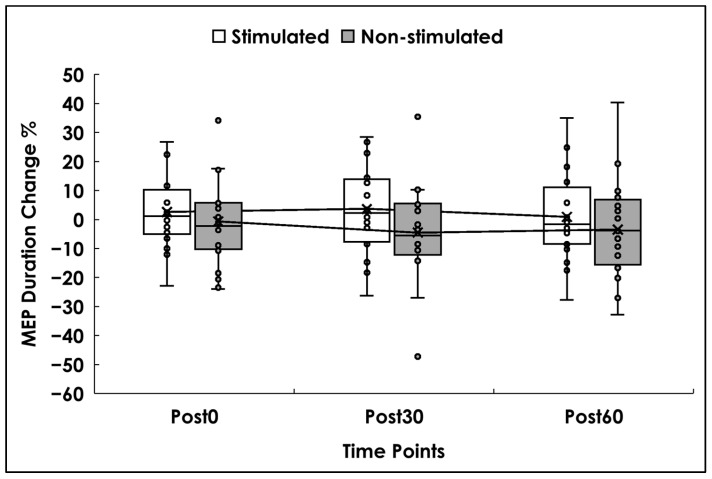
MEP Duration. Box and whisker plots represent the percentage change in MEP duration values (%), for the stimulated (white) and non-stimulated sides (gray) at 0 min (Post0), 30 min (Post30), and 60 min (Post60) after the stimulation. Small dots represent individual data, while the lower and upper horizontal lines represent minimum and maximum values. Boxes range from the 1st to the 3rd quartile, the X marks the mean values, and the middle horizontal lines represent median values. A mean line illustrates the trajectory of MEP duration across Post0, Post30, and Post60.

Analyses were conducted on 19 participants. There was no significant main effect of side (F(1,18) = 1.860, *p* = 0.189, ηp^2^ = 0.094), time (F(2,36) = 1.05, *p* = 0.361, ηp^2^ = 0.055), or the time × side interaction (F(2,36) = 0.90, *p* = 0.417, ηp^2^ = 0.047). Descriptively, duration on the stimulated side increased modestly at Post0 (3%) and Post 30, while the non-stimulated side showed decreases of (1–4%) across time points (see Figure 6).

## 4. Discussion

This study aimed to determine the temporal effectiveness of rPMS on CSE of the TA muscle in healthy adults, as reflected by changes in MEP amplitude, duration, and latency, comparing the stimulated TA with its non-stimulated counterpart in healthy adults. Our findings indicate that rPMS has the potential to facilitate CSE in the stimulated TA muscle relative to the non-stimulated side. This was reflected in a significant difference in normalized peak-to-peak MEP amplitude between sides, although no corresponding effects were observed for MEP latency or MEP duration. Together, these findings provide partial support for our hypothesis, indicating side-specific facilitation of CSE without consistent temporal modulation across all MEP parameters.

We observed a modest increase in normalized peak-to-peak MEP amplitude in the stimulated TA muscle compared to the non-stimulated side. This finding is generally in line with prior studies showing that a single session of rPMS can enhance CSE in upper-limb muscles [5,6,7]. In this study, we used a stimulation protocol involving rPMS at 20 Hz for 15 min at ~10% above motor threshold, based on prior research showing that stimulation in the 20–50 Hz range for 10–20 min at similar intensities can elicit neuroplastic changes in healthy adults [5,6].

The effects on MEP amplitude are consistent with previous studies reporting increased normalized MEP amplitude [7], peak MEP amplitude [6], and steeper MEP recruitment curves after rPMS [5,6]. Some studies have also shown that rPMS can enhance intracortical facilitation in upper-limb muscles such as the FCR and ECR, though no changes in spinal excitability (measured by M-wave or H-reflex) were reported [5,6,7]. These findings suggest that rPMS may promote cortical and intracortical neuroplastic changes, likely through the transmission of proprioceptive input that modulates excitatory interneuron activity via glutamatergic pathways and possibly through LTP-like mechanisms [2,4].

The observed difference between hemispheres may also reflect the influence of rPMS on interhemispheric communication via the corpus callosum, potentially facilitating CSE on the contralateral side while reducing excitability ipsilaterally. However, this remains speculative, as interhemispheric inhibition was not directly assessed in this study.

Although not statistically significant, a descriptive increase in MEP amplitude was observed at 30 min post-stimulation. This temporal pattern should be interpreted cautiously, as the overall time effect was not significant. Similar descriptive temporal patterns have been reported in previous rPMS studies, including observations of relatively greater responses at 30 min post-stimulation [17]. These findings are consistent with reports from upper-limb studies, in which facilitation peaked at intermediate time points and gradually diminished thereafter [5,6,7]. It is possible that immediate post-stimulation effects were masked by transient fatigue induced by the repetitive contractions during rPMS [18]. A short rest period may have allowed recovery by the 30 min mark, temporarily enhancing responsiveness before effects waned again at 60 min.

For MEP latency, we did not observe significant changes across time or between the sides. MEP latency primarily reflects the conduction time of descending corticospinal volleys from the motor cortex to spinal motoneurons [3]. The absence of latency modulation may indicate that the rPMS protocol employed did not sufficiently alter corticospinal conduction speed or synaptic transmission timing, indicating that rPMS-induced effects may preferentially influence excitability magnitude rather than transmission velocity. In addition, MEP latency is known to exhibit relatively low sensitivity to neuromodulatory interventions and may be influenced by individual factors such as height, age, or sex, which may also have contributed to the null findings.

Similarly, rPMS did not significantly affect MEP duration. MEP duration reflects the temporal summation of neural inputs arising from cortical, subcortical, and spinal sources. Prior evidence suggests that MEP duration is particularly sensitive to propriospinal and spinal interneuronal activity rather than purely cortical mechanisms [7,19]. Given that rPMS is thought to primarily influence afferent-driven modulation at the cortical level, this may explain the lack of observable change in this measure [7,19].

Moreover, these findings can be interpreted within the broader literature on TMS-based neuromodulatory protocols that exploit peripheral afferent input to modulate CSE through LTP-like mechanisms. Paired Associative Stimulation (PAS), for example, combines peripheral electrical stimulation with time-based single-pulse TMS delivered over M1. This associative pairing is thought to induce spike-timing-dependent plasticity, resulting in LTP-like facilitation of the corticospinal pathways [20]. In contrast, rPMS delivers repetitive magnetic pulses exclusively at the peripheral level, evoking rhythmic muscle contractions and sustained proprioceptive afferent input. Such afferent drive may indirectly modulate corticospinal excitability by engaging excitatory sensorimotor circuits through LTP-like plastic mechanisms [2,4]. Consistent with this framework, the present findings demonstrated that increased MEP amplitude following rPMS aligns with previous PAS studies reporting enhanced MEP amplitude in the healthy TA muscle [21].

Several limitations should be acknowledged. The relatively large standard deviations observed reflect inter- and intra-individual variability inherent to MEP measurements, which may have reduced sensitivity to detect subtle effects, and this variability could stem from both intrinsic (e.g., fluctuations in cortical excitability) and extrinsic (e.g., coil positioning, EMG noise) factors [12,22]. The absence of behavioral or functional measures limits the interpretation of physiological changes. In addition, the stimulation parameters employed may not represent the most effective combination for modulating lower limb CSE, and covariate analyses based on sex or age were not performed. Further limitations include the relatively small sample size, the high rate of data exclusion, causing different analyzable samples across outcomes resulting from outlier handling, which may limit direct comparability between MEP metrics, and the lack of a sham stimulation control group, which together may limit generalizability and the ability to fully isolate stimulation-specific effects. Finally, although multiple neurophysiological markers were examined, future studies may benefit from incorporating additional measures, such as the cortical silent period or interhemispheric inhibition, to further elucidate the mechanisms underlying rPMS-induced modulation of CSE.

## 5. Conclusions

In conclusion, a single session of rPMS applied to the tibialis anterior muscle resulted in a significant difference in CSE between stimulated and non-stimulated sides, as indicated by MEP amplitude. While the time course of this effect was not statistically significant, a peak at 30 min post-stimulation was observed. No effects were found for MEP latency or duration. These findings contribute to the limited literature on rPMS in the lower limbs and suggest that rPMS may modestly enhance CSE under specific conditions. Further research is needed to optimize stimulation parameters and explore their relevance in clinical populations.

## Figures and Tables

**Table 1:** Participant characteristics.

Demographics	Participants, n = 20
Age (Years)	25.5 (5.4)
Sex (Females/Males)	(11/9)
Race/Ethnicity	
White, not Hispanic or Latino	9 (45%)
White, Hispanic or Latino	0 (0%)
Asian American	11 (55%)
African American	0 (0%)
Dominant side (Right/Left)	(20/0)

General characteristics of participants. n: number. Values are count (percentage), except for age as mean (SD).

**Table 2:** Descriptive statistics for motor evoked potential (MEP) parameters.

Time Point	Side	Amplitude (mV)	Change %	Latency (ms)	Change %	Duration (ms)	Change %
Pre	Stimulated	0.83 (0.4)	-	24.1 (3.5)	-	41.0 (8.1)	-
	Non- stimulated	0.76 (0.4)	-	24.2 (1.6)	-	39.8 (6.1)	-
Post0	Stimulated	0.88 (0.4)	8.9 (21.5)	23.2 (3.0)	−1.8 (8.3)	41.5 (6.9)	2.6 (12.3)
	Non- stimulated	0.75 (0.3)	−8.7 (10.9)	24.7 (2.2)	2.6 (8.2)	39.4 (7.5)	−0.6 (16.7)
Post30	Stimulated	0.89 (0.3)	12.0 (27.3)	23.1 (3.1)	−3.3 (7.2)	41.9 (8.0)	3.6 (15.5)
	Non- stimulated	0.75 (0.3)	−7.2 (13.1)	24.3 (2.6)	0.4 (8.8)	38.1 (8.9)	−4.5 (16.3)
Post60	Stimulated	0.89 (0.3)	7.1 (16.0)	23.5 (3.4)	−1.6 (11.3)	40.7 (7.8)	−0.9 (13.2)
	Non- stimulated	0.74 (0.3)	−10.4 (16.0)	24.5 (2.6)	1.3 (9.4)	38.5 (9.2)	−3.4 (17.1)

Values are presented in mean (SD) for stimulated and non-stimulated sides at each time point. Change % represents the percentage change in the initial baseline measurements.

## Data Availability

The data presented in this study are available on request from the corresponding author due to ethical and institutional regulations.

## Abbreviations

The following abbreviations are used in this manuscript:

rPMS	Repetitive Peripheral Magnetic Stimulation
rTMS	Repetitive Transcranial Magnetic Stimulation
CSE	Corticospinal Excitability
LTP	Long-Term Potentiation
MEP	Motor Evoked Potential
APB	Abductor Pollicis Brevis
FCR	Flexor Carpi Radialis
ECR	Extensor Carpi Radialis
TA	Tibialis Anterior
TMS	Transcranial Magnetic Stimulation
MVC	Maximum Voluntary Contraction
EMG	Electromyography
M1	Primary Motor Cortex
AMT	Active Motor Threshold
PAS	Paired Associative Stimulation
